# Hepatocyte TrkB Acts as a Gatekeeper Against MASH‐Related Liver Fibrosis by Suppressing the TGFβ/CCL2 Axis and Macrophage Infiltration

**DOI:** 10.1111/cpr.70202

**Published:** 2026-04-03

**Authors:** Yueying Chen, Jiayi Wei, Shuxuan Li, Kefan Yin, Heming Wang, Yicheng Zhao, Shuqiang Weng, Xizhong Shen, Guangqi Song, Changfeng Zhu, Qunyan Yao, Ling Dong

**Affiliations:** ^1^ Department of Gastroenterology and Hepatology Zhongshan Hospital, Fudan University Shanghai China; ^2^ Shanghai Institute of Liver Diseases Shanghai China; ^3^ Department of Oncology Renji Hospital, Shanghai Jiao Tong University School of Medicine Shanghai China; ^4^ Department of Gastroenterology Huadong Hospital, Shanghai Medical College, Fudan University Shanghai China; ^5^ Joint Laboratory of Biomaterials and Translational Medicine, Puheng Biomedicine Co., Ltd Shanghai China; ^6^ Chinese Medicine Guangdong Laboratory/State Key Laboratory of Traditional Chinese Medicine Syndrome, The Second Affiliated Hospital of Guangzhou University of Chinese Medicine (Guangdong Provincial Hospital of Chinese Medicine), Guangzhou University of Chinese Medicine Guangzhou China; ^7^ Department of Gastroenterology and Hepatology Zhongshan Hospital (Xiamen), Fudan University Xiamen China; ^8^ Shanghai Geriatric Medical Center Shanghai China

**Keywords:** 3D model, liver fibrosis, MoMFs recruitment, TrkB

## Abstract

Liver fibrosis represents a critical pathological stage in chronic liver disease, characterized by excessive activation of hepatic stellate cells (HSCs) and dysregulated immune cell recruitment. Our previous studies demonstrated that overexpressing tyrosine kinase receptor B (TrkB) in HSCs inhibits their activation, thereby alleviating liver fibrosis. However, its functional significance in hepatocytes—the predominant parenchymal cells orchestrating liver homeostasis—remains poorly understood. Here, we investigate the mechanistic interplay between hepatocyte‐specific TrkB signalling and liver fibrosis progression. Through integrated in vivo animal models and in vitro two‐ and three‐dimensional systems, we demonstrate that elevated TrkB expression in hepatocytes reduces pro‐fibrotic and inflammatory cytokines, attenuates HSC activation via paracrine signalling, and impairs monocyte‐derived macrophage (MoMF) recruitment. Mechanistically, TrkB modulates the TGFβ/SMAD3 pathway by inhibiting p‐SMAD3 nuclear translocation, thus suppressing FOS transcription. As a core component of the AP‐1 transcription factor complex, FOS directly regulates CCL2, a pivotal chemokine for macrophage recruitment. Collectively, these results establish TrkB as a key regulatory node in the TGFβ/SMAD3/FOS/CCL2 signalling cascade, orchestrating macrophage‐mediated fibrotic responses in the liver.

## Graphical abstract

Hepatocyte TrkB is identified as a critical gatekeeper against MASH‐related fibrosis. Mechanistically, TrkB inhibits the TGFβ/SMAD3/FOS axis to suppress CCL2 secretion, thereby blocking pathogenic macrophage recruitment and ameliorating liver fibrosis.

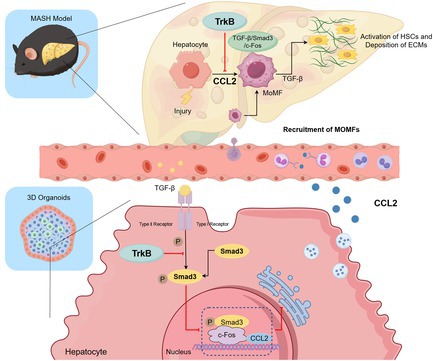

**Abbreviations**
AAV8: adeno‐associated virus vector serotype 8
ACTA2: actin alpha 2
AKT: protein kinase B
ANOVA: analysis of variance
AP‐1: activator protein 1
BDNF: brain‐derived neurotrophic factor
CCL2: C‐C motif chemokine ligand 2
CCl_4_
: carbon tetrachloride
CCR2: C‐C motif chemokine receptor 2
CD11b: integrin alpha M
CDKNIA: cyclin dependent kinase inhibitor 1A
CDS: coding domain sequence
ChIP: chromatin immunoprecipitation
COL1A1: collagen type I alpha 1
CREB: cAMP response element binding protein
DEGs: differentially expressed genes
DMEM: Dulbecco's modified Eagle's
ECM: extracellular matrix
Erk: extracellular regulated kinase
F4/80: adhesion G protein‐coupled receptor E1
FOS: Fos proto‐oncogene
FXR: Farnesoid X receptor
GAN: Gubra‐Amylin MASH
H&E: haematoxylin and eosin
hnRNP U: heterogeneous nuclear ribonucleoprotein U
HSCs: hepatic stellate cells
IL‐6: Interleukin‐6
KCs: Kupffer cells
MASH: metabolic dysfunction‐associated steatohepatitis
MFs: myofibroblasts
MoMFs: monocyte‐derived macrophages
MYC: MYC proto‐oncogene
NAC: nucleic acid
PA: palmitic acid
PBMCs: peripheral blood mononuclear cells
qRT‐PCR: quantitative reverse transcriptase polymerase chain reaction
RANKL: receptor activator of nuclear factor‐KB ligand
SEM: standard error of mean
SMAD3: SMAD family member 3
STAT1: signal transducer and activator of transcription 1
TGFβ: transforming growth factor β
TNFα: tumour necrosis factor‐alpha
TrkB: tyrosine kinase receptor B

## 1 Introduction

Chronic liver disease is a major global health issue, causing over two million deaths annually. Liver fibrosis, a critical stage in its progression, involves a cascade of events starting with hepatocyte injury [1]. This injury activates inflammatory cells and triggers the subsequent activation of hepatic stellate cells (HSCs) and myofibroblasts (MFs), leading to excessive secretion of extracellular matrix (ECM) [2]. Macrophages, including resident Kupffer cells (KCs) and recruited monocyte‐derived macrophages (MoMFs), further exacerbate the inflammatory response and fibrosis [3, 4]. Recent investigations into this process have elucidated several molecular pathways, including those modulated by TGFβR I inhibition (pirfenidone), FXR activation (obeticholic acid), and CCL24 neutralization (CM‐101) [5, 6, 7, 8]. However, the intricate network of cellular and molecular interactions underlying fibrogenesis remains incompletely characterized, highlighting the need for continued mechanistic exploration of this multifaceted pathological process.

TrkB, a receptor tyrosine kinase from the tyrosine kinase family, is distinguished by its extracellular glycosylated polypeptide, transmembrane domain, and intracellular tyrosine kinase region [9]. In the liver, TrkB is crucial for neuronal support and hepatic haematopoiesis. Mutations in TrkB have been linked to increased food intake, abnormal obesity, and liver steatosis in both humans and mice, suggesting a potential involvement in chronic liver disease [10, 11, 12]. Our prior research showed that overexpression of TrkB in HSCs establishes a negative feedback loop with the TGFβ/SMADs pathway, effectively inhibiting HSCs proliferation and activation, thereby mitigating liver fibrosis [13]. Intriguingly, emerging evidence suggests TrkB may exert broader regulatory functions in hepatocytes. Research indicates that deficiency in heterogeneous nuclear ribonucleoprotein U (hnRNP U) leads to increased expression of a truncated TrkB isoform (TrkB‐T1), which exacerbates inflammatory signalling and stress‐induced cell death in hepatocytes [14]. Given the essential role of hepatocytes in liver function and their predominant population compared to HSCs, targeting these cells for TrkB overexpression offers substantial advantages. Contemporary advances in cell‐specific delivery platforms—spanning lipid‐based nanoparticles, viral vectors, and GalNAc‐conjugated systems—have enabled precise investigation of hepatocyte‐specific molecular manipulations [15, 16, 17]. These hepatocyte‐focused systems enable mechanistic studies of TrkB in MASH‐related fibrosis, revealing how hepatocyte TrkB signalling interacts with metabolic stress pathways during fibrotic remodelling [18].

In this study, we aim to explore the role of TrkB overexpression in hepatocytes on the progression of MASH related liver fibrosis. To elucidate the interactions between hepatocytes and other fibrosis‐related cells, we employed in vitro 2D and 3D models alongside animal models. Our findings revealed that TrkB overexpression in hepatocytes significantly reduces the expression and secretion of inflammatory and fibrogenic cytokines, while also inhibiting the recruitment and infiltration of MoMFs into the liver, leading to a reduction in fibrosis progression. Further analysis showed that TrkB overexpression in hepatocytes inhibits the TGFβ/SMAD3 pathway, resulting in the downregulation of the transcription factor FOS and subsequent suppression of CCL2 expression and secretion, which serves as a key regulator in macrophage infiltration during fibrosis progression. These results indicate that TrkB overexpression in hepatocytes may attenuate macrophage recruitment, thereby mitigating liver fibrosis by modulating the TGFβ/SMAD3/FOS/CCL2 axis.

## 2 Results

### 2.1 Hepatocyte TrkB Expression Levels Correlate With MASH‐Related Fibrosis Progression

In our previous research, both in vivo and in vitro studies demonstrated that overexpression of TrkB in HSCs alleviates fibrosis by inhibiting the TGFβ/SMAD3 pathway, highlighting TrkB's potential as a target for anti‐fibrotic therapy [13]. However, since those studies primarily focused on TrkB expression in HSCs, it became crucial to investigate whether similar effects could be observed in the broader liver context, particularly in relation to hepatocytes.

**FIGURE 1:**
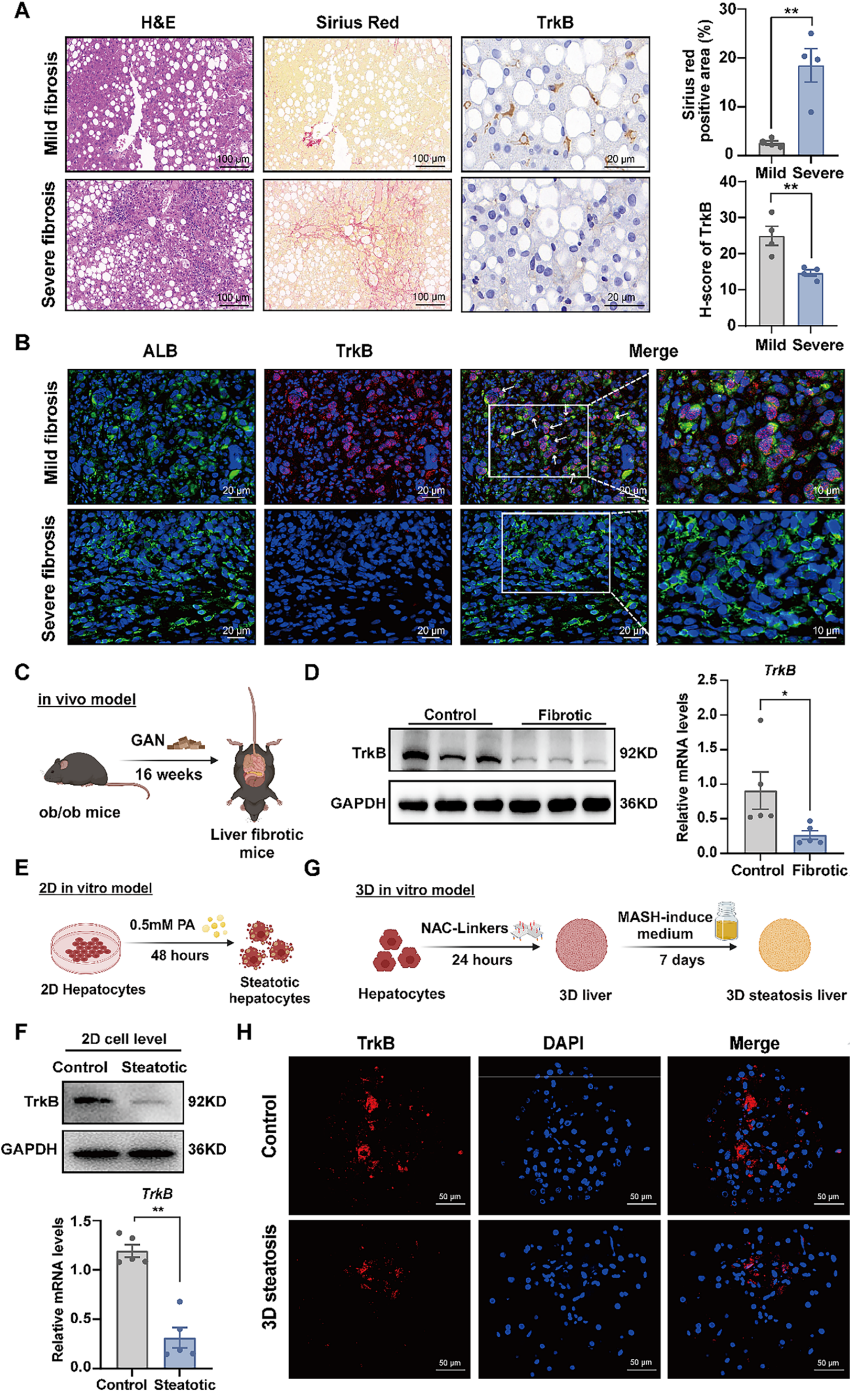
Hepatocyte TrkB expression levels correlate with MASH‐related fibrosis progression. (A) Representative images and quantification of H&E, Sirius Red and IHC of TrkB in mild (S0‐1) and severe fibrosis (≥ S2) patients with MASH‐related fibrosis, the grading of fibrosis is based on the Scheuer scoring system. Scale bars, 100 μm (*n* = 4 per group). (B) TrkB and albumin co‐immunofluorescence in liver samples from MASH‐related fibrosis patients with mild (S0‐1) and severe fibrosis (≥ S2). (C) Schematic overview of GAN‐fed ob/ob mouse model. (D) The protein level (left) and expression level (right) of hepatocyte TrkB were detected by Western blot analysis (*n* = 3 per group) and qRT‐PCR (*n* = 4 per group) in primary hepatocytes isolated from control and GAN‐fed ob/ob mouse. (E) Schematic overview of PA‐treated hepatocyte model. Schematic overview of PA‐treated hepatocyte model. (F) The protein level (left) and expression level (right) of TrkB were detected by Western blot analysis (*n* = 3 per group) and qRT‐PCR (*n* = 5 per group) in control and PA‐treated hepatocytes. (G) Schematic overview of the 3D model was constructed by human primary hepatocytes in a total of 3000 cells per NACs model. (H) Representative images of immunofluorescence staining of TrkB in 3D control and liver steatosis model. Scale bars: 50 μm. Data were mean ± SEM (*p < 0.05, **p < 0.01, ***p < 0.001; Student's *t*‐test). Schematic illustration (C, E, G) was created by biorender.

To address this, we analysed TrkB expression in liver tissues from patients with MASH‐related fibrosis. Notably, patients with severe fibrosis exhibited significantly lower TrkB levels compared to those with mild fibrosis, suggesting that TrkB downregulation is associated with disease progression (Figure 1A and Figure S1A). Building on these findings, we further explored TrkB expression changes specifically in hepatocytes. To systematically investigate the role of TrkB in MASH‐related fibrosis, we first employed a GAN‐fed ob/ob mouse model, characterized by leptin deficiency and replicating the metabolic disturbances observed in human MASH progression (Figure 1C). Using PCR and western‐blot analysis of primary hepatocytes isolated from these mice, we confirmed a reduction of TrkB at both mRNA and protein levels during MASH progression (Figure 1D).

To complement these findings, we established two complementary in vitro systems: a conventional 2D hepatocyte culture and an advanced 3D liver steatosis model. In the 2D system, primary hepatocytes exposed to palmitic acid (PA)—a saturated free fatty acid widely used to induce lipotoxicity and fibrotic responses—exhibited a marked reduction in TrkB mRNA and protein expression (Figure 1E,F), consistent with our in vivo observations. To better mimic the pathophysiological microenvironment of the liver, we leveraged a 3D hepatocyte‐only culture system engineered via the Nucleic‐Acid‐nanostructures‐decorated‐living‐Cells (NACs) strategy, which preserves cell–cell interactions and enhances metabolic fidelity [19] (Figure 1G). Similarly, this 3D model also demonstrated a pronounced decrease in TrkB expression upon induction of steatosis (Figure 1H). This uniform downregulation of TrkB across different models underscores its critical role in the progression of MASH‐related liver fibrosis. Notably, while previous studies revealed contrasting trends in TrkB expression in HSCs at the mRNA and protein levels during MASH progression, the synchronized downregulation of TrkB in hepatocytes suggests a distinct role for TrkB in these cells, contributing uniquely to liver fibrosis progression. These results highlight hepatocyte TrkB as a key molecular player in MASH‐driven fibrosis and reinforce its potential as a therapeutic target for mitigating liver fibrosis.

### 2.2 Hepatocyte‐Specific Overexpression of TrkB Ameliorates Hepatic Inflammation and Fibrosis

**FIGURE 2:**
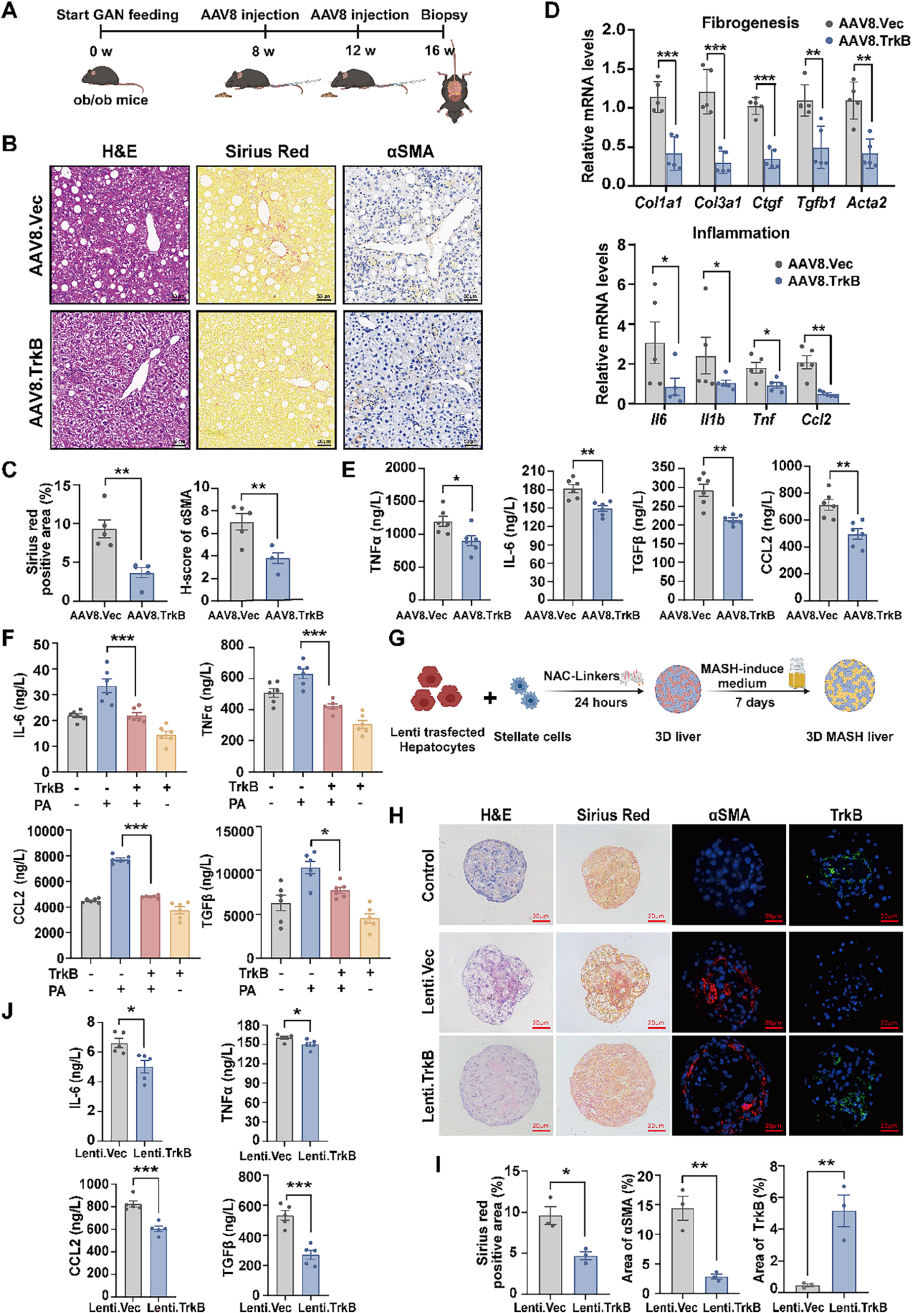
Hepatocyte‐specific overexpression of TrkB ameliorates hepatic inflammation and fibrosis. (A) Schematic diagram of the AAV8‐injected animal model experiment. (B) Representative images of H&E, Sirius Red and immunohistochemical staining (IHC) of αSMA in GAN‐fed ob/ob liver samples of AAV8‐control and AAV8‐TrkB mice. Scale bars, 50 μm. (C) Quantification of Sirius Red staining and H‐score values of αSMA in (B) (*n* = 5 per group). (D) qRT‐PCR analysis of *Col1a1, Col3a1, Ctgf, Tgfb, Acta2, IL‐6, IL‐1b, Tnfα*, and *Ccl2* in GAN‐fed ob/ob liver samples of AAV8‐control and AAV8‐TrkB mice (*n* = 5 per group). (E) ELISA analysis of IL‐6, TNFα, CCL2 and TGFβ concentrations in GAN‐fed ob/ob blood serum samples of AAV8‐control and AAV8‐TrkB mice (*n* = 6 per group). (F) ELISA analysis of IL‐6, TNFα, CCL2 and TGFβ concentrations in the supernatant of control and PA‐treated HepG2 and HepG2‐TrkB (*n* = 6 per group). (G) Schematic overview of 3D MASH liver construction. The 3D model was constructed by PHHs and HSCs in a total of 3000 cells per NACs model. PHHs: HSCs = 10:1. (H) Representative images of H&E, Sirius Red and immunofluorescence of αSMA and TrkB in control, lenti‐vector and lenti‐TrkB 3D MASH livers. (I) Quantification of Sirius Red staining and immunofluorescence of αSMA and TrkB in (G) (*n* = 3 per group). (J) ELISA analysis of IL‐6, TNFα, CCL2 and TGFβ concentrations in the supernatant of lenti‐vector and lenti‐TrkB 3D MASH livers (*n* = 5 per group). Data were mean ± SEM (*p < 0.05, **p < 0.01, ***p < 0.001; Student's *t*‐test in C–E, J, I; One way ANOVA in F). Schematic illustration (A) and (G) were created by biorender.

To determine whether hepatocyte‐specific TrkB modulation influences liver fibrosis progression, we employed an AAV8 vector to overexpress TrkB in hepatocytes of GAN‐fed ob/ob mice (Figure 2A). Mice injected with AAV8‐TrkB showed significant improvements in liver fibrosis compared to the AAV8‐GFP‐injected control group (Figure 2B,C). Moreover, the expression of genes associated with inflammation and fibrosis was notably decreased (Figure 2D), accompanied by reduced secretion of pro‐inflammatory cytokines and fibrogenic factors such as IL‐6, TNFα, CCL2, and TGFβ (Figure 2E). These findings collectively suggest that hepatocyte TrkB may regulate inflammatory and fibrogenic signalling cascades.

Hepatic cell injury drives and perpetuates liver fibrosis through key mediators, such as inflammatory cytokines (IL‐6, TNFα, CCL2) and fibrogenic factors (TGFβ). In light of the reduced cytokine levels observed in the AAV8‐TrkB mouse model, we next investigated whether hepatocyte TrkB modulates the secretion of inflammatory factors. Using a conditioned medium approach (Figure S2A), we found that TrkB‐overexpressing hepatocytes, when stimulated with palmitic acid (PA), showed a suppression of inflammatory and profibrotic gene expression (Figure 2F and Figure S3A). This was accompanied by reduced cytokine secretion, which significantly inhibited HSC proliferation and decreased the expression of fibrotic markers, such as COL1A1 and α‐SMA (Figures S2B–D and S3B).

To overcome the limitations of 2D cell models, we developed a more physiologically relevant 3D multi‐cell liver MASH NACs model, incorporating lentivirus‐transfected primary human hepatocytes and HSCs (Figure 2G). In this model, TrkB overexpression in hepatocytes resulted in reduced secretion of inflammatory cytokines and TGFβ, ultimately alleviating liver fibrosis (Figure 2H–J). Collectively, these findings highlight that TrkB overexpression in hepatocytes effectively reduces the secretion of inflammatory and profibrotic factors, thereby mitigating liver fibrosis.

### 2.3 Hepatocyte TrkB Overexpression Suppresses Fibrosis by Modulating Macrophage Recruitment via CCL2/CCR2 Axis

**FIGURE 3:**
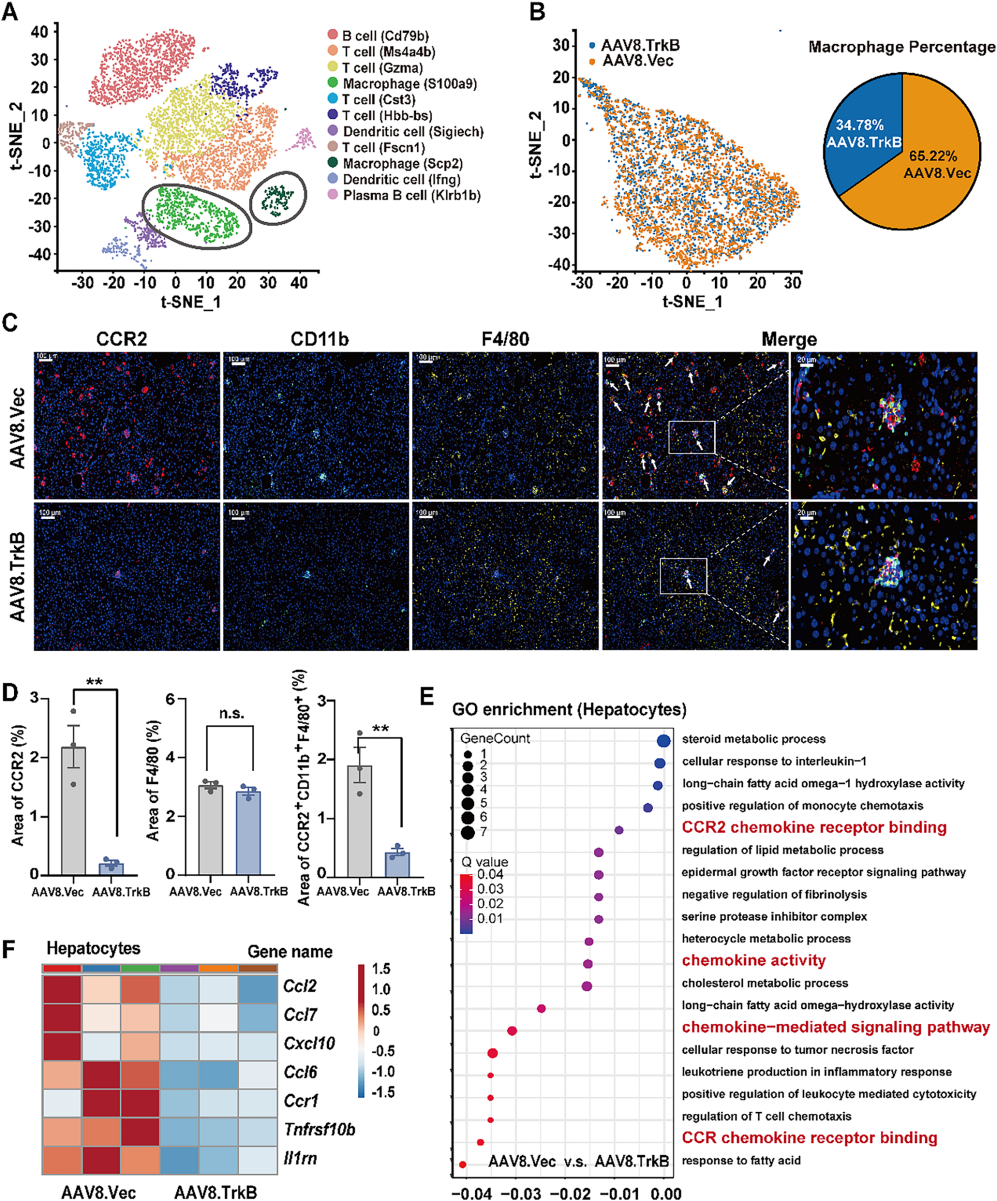
Hepatocyte‐specific overexpression of TrkB attenuates recruitment of MoMFs by restraining CCL2/CCR2 axis. (A) T‐SNE plot of scRNA‐seq on immune cells sorted from GAN‐fed ob/ob liver samples of AAV8‐control and AAV8‐TrkB mice. (B) T‐SNE plot and the percentage of macrophages from GAN‐fed ob/ob liver samples of AAV8‐control and AAV8‐TrkB mice (*n* = 2 per group). (C) CCR2, CD11b and F4/80 co‐immunofluorescence in liver samples from AAV8‐control and AAV8‐TrkB mice. The white arrows indicate the CCR2 + CD11b + F4/80+ region. Scale bars, 100 μm. (D) Quantification of CCR2, F4/80 and the co‐positive area for three markers in (C) (*n* = 3 per group). (E) GO enrichment analysis of RNA‐seq from mouse primary hepatocytes isolated from GAN‐fed ob/ob mice of AAV8‐control and AAV8‐TrkB group (*n* = 3 per group). (F) Gene expressions related to inflammation and macrophage recruitment from mouse primary hepatocytes isolated from GAN‐fed ob/ob mice of AAV8‐control and AAV8‐TrkB group detected by RNA‐seq (*n* = 3 per group). Data were mean ± SEM (*p < 0.05, **p < 0.01, ***p < 0.001; Student's *t*‐test).

Given that hepatocyte TrkB overexpression suppresses inflammatory progression in hepatocytes, we recognized that hepatic inflammatory responses are coordinately regulated by multiple immune cell populations within the liver microenvironment. To systematically characterize hepatocyte TrkB‐mediated immunomodulation, we performed single‐cell RNA sequencing of liver immune cells. This analysis revealed a markedly reduced macrophage proportion in AAV8‐TrkB‐injected mice (Figure 3A,B). Immunofluorescence co‐localization with CCR2, CD11b, and F4/80 markers further indicated that, while the F4/80 fluorescence intensity slightly decreased in AAV8‐TrkB‐injected mice (without statistical significance), there was a notable reduction in both the fluorescence area of CCR2 and the triple‐positive area of CCR2, CD11b, and F4/80. This suggests that hepatocyte‐specific TrkB overexpression inhibits the recruitment and infiltration of MoMFs, typically indicated by CCR2^+^CD11b^+^F4/80^+^ (Figure 3C,D). This finding is consistent with the increased prevalence of CD68^+^ CCR2^+^ cells in patients with severe liver fibrosis compared to those with mild fibrosis (Figure S4A,B). Therefore, the anti‐fibrotic effects of TrkB likely arise from both its direct impact on HSC activation and its modulation of the immune microenvironment.

To investigate how hepatocytic TrkB influences MoMFs recruitment, we performed RNA sequencing on primary hepatocytes isolated from mice, revealing significant enrichment in pathways related to CCR2 receptor binding and chemokine signalling. Notably, several inflammatory chemokines, particularly CCL2, were downregulated in the TrkB overexpression group (Figure 3E,F and Figure S5A). Given the established role of the CCL2/CCR2 axis in macrophage recruitment, these results indicate that hepatocytic TrkB overexpression may inhibit MoMFs infiltration by downregulating this signalling pathway.

**FIGURE 4:**
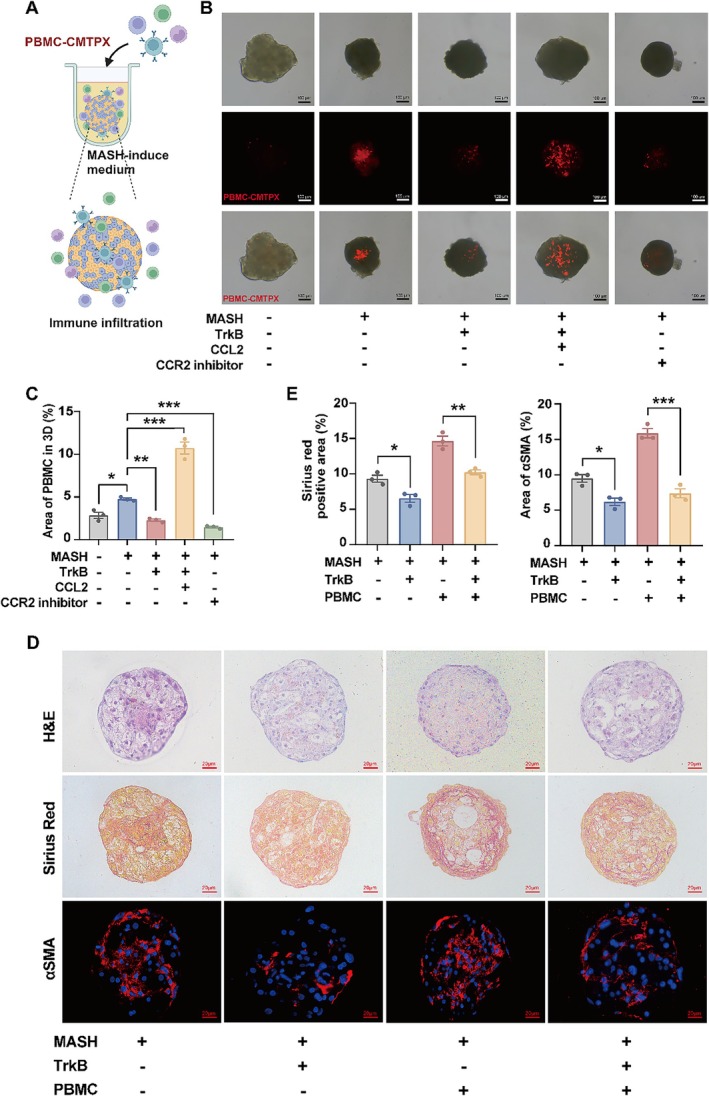
Hepatocyte‐specific overexpression of TrkB attenuates recruitment of MoMFs by restraining CCL2/CCR2 axis. (A) Schematic overview of 3D MASH livers with immune cell infiltration construction. The 3D model was constructed by PHHs and HSCs in a total of 3000 cells per NACs model. PHHs: HSCs = 10:1. PBMCs were pre‐stained with CMTPX live cell tracker before adding to the culture environment. (B) Representative images of PBMC infiltration in 3D livers. (C) Quantification of PBMC infiltration in (B) (*n* = 3 per group). (D) Representative images of Sirius Red and IF of αSMA in 3D MASH livers. (E) Quantification of Sirius Red staining, and αSMA in (L) (*n* = 3 per group). Data were mean ± SEM (*p < 0.05, **p < 0.01, ***p < 0.001; One way ANOVA). Schematic illustration (A) was created by biorender.

To further confirm TrkB's role in macrophage recruitment, we developed a 3D MASH NACs model that included hepatocytes and HSCs, along with immune cell infiltration (Figure 4A). In this model, we introduced CMTPX‐labelled peripheral blood mononuclear cells (PBMCs) to track their infiltration. TrkB overexpression resulted in reduced CCL2 secretion within the 3D model (Figure S5B), which in turn inhibited PBMCs' infiltration. Notably, this effect was reversed with the addition of CCL2 (Figure 4B,C), confirming that TrkB modulates macrophage recruitment through the CCL2/CCR2 axis in the context of liver fibrosis. Furthermore, while the presence of PBMCs in 3D models significantly exacerbated fibrotic changes, hepatocyte‐specific TrkB overexpression demonstrated a markedly stronger therapeutic effect on HSC activation and proliferation compared to the control group (Figure 4D,E). This highlights the critical role of immune cell recruitment and infiltration in mediating the anti‐fibrotic effects of TrkB.

### 2.4 TrkB Overexpression in Hepatocytes Mitigates Fibrosis by Inhibiting CCL2 Secretion via TGFβ/SMAD3/FOS Pathway

**FIGURE 5:**
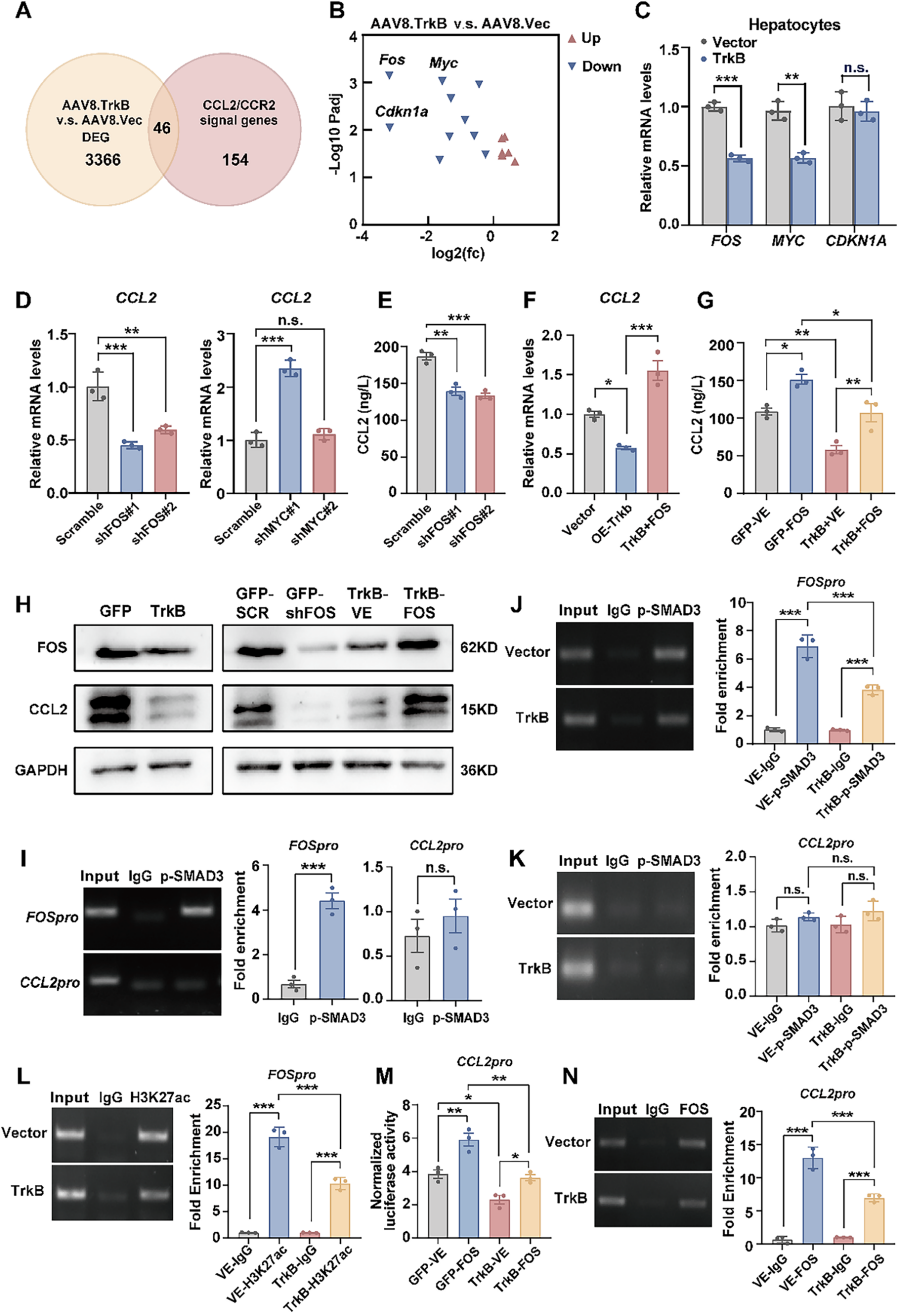
Hepatocytic TrkB overexpression suppresses CCL2 secretion via TGF‐β/SMAD3/FOS axis. (A) Venn diagram analysis of the intersection with differentially expressed genes (DEGs) in AAV8 control and AAV8‐TrkB mice livers, and the genes involved in the TGFB/SMAD3 pathways. (B) Volcano plot showing the top 16 most significantly altered DEGs identified in (A) between AAV8‐control and AAV8‐TrkB mice livers. (C) qRT‐PCR analysis of *FOS, MYC*, and *CDKN1A* in HepG2 and HepG2‐TrkB cells (*n* = 3 per group). (D) Relative expression of *CCL2* detected in control, HepG2‐shFOS and shMYC cells (*n* = 3 per group). (E) ELISA analysis of CCL2 concentration in control and HepG2‐shFOS cells (*n* = 3 per group). (F) Relative *CCL2* expression level detected by qRT‐PCR in HepG2, HepG2‐TrkB and HepG2‐TrkB‐FOS cells (*n* = 3 per group). (G) ELISA analysis of CCL2 concentration in HepG2, HepG2‐shFOS, HepG2‐TrkB and HepG2‐TrkB‐FOS cells (*n* = 3 per group). (H) Western blot analysis showed the protein levels of FOS and CCL2 in HepG2, HepG2‐shFOS, HepG2‐TrkB and HepG2‐TrkB‐FOS cells. (I) CHIP‐qPCR analysis of p‐SMAD3 binding to *FOS* and *CCL2* promoters in HepG2 cells (*n* = 3 per group). (J) The binding of p‐SMAD3 to *FOS* promoter in HepG2 and HepG2‐TrkB cells were detected by CHIP‐qPCR analysis (*n* = 3 per group). (K) The binding of p‐SMAD3 to *CCL2* promoter in HepG2 and HepG2‐TrkB cells were detected by CHIP‐qPCR analysis (*n* = 3 per group). (L) CHIP‐qPCR analysis showed the binding of H3K27ac to *FOS* promoter in HepG2 and HepG2‐TrkB cells (*n* = 3 per group). (M) Relative luciferase activity of *CCL2* promoter in HepG2, HepG2‐FOS, HepG2‐TrkB and HepG2‐TrkB‐FOS cells was detected by Luciferase reporter gene assay (*n* = 3 per group). (N) CHIP‐qPCR analysis of FOS binding to *CCL2* promoter in HepG2, HepG2‐FOS, HepG2‐TrkB and HepG2‐TrkB‐FOS cells (*n* = 3 per group). Data were mean ± SEM (*p < 0.05, **p < 0.01, ***p < 0.001; Student's *t*‐test in C, I; One way ANOVA in D–G, J–N).

To elucidate the molecular mechanisms by which TrkB overexpression reduces CCL2 secretion in hepatocytes, we conducted a comprehensive analysis of differentially expressed genes (DEGs) between the livers of AAV8‐TrkB and AAV8‐GFP‐injected mice. By intersecting these DEGs with genes associated with the CCL2/CCR2 signalling pathway, we identified 46 genes with a *p* < 0.05. Among these, FOS, MYC, and CDKN1A emerged as the most significantly altered genes (Figure 5A,B). In vitro experiments corroborated these findings, demonstrating that TrkB overexpression significantly reduced the mRNA levels of FOS and MYC (Figure 5C). Notably, both FOS and MYC overexpression upregulated CCL2 expression, while FOS knockdown downregulated it (Figure 5D,E and Figure S6A). In contrast, MYC knockdown showed no consistent effect on CCL2 levels (Figure 5D). Importantly, TrkB overexpression reduced both FOS and CCL2 levels in hepatocytes, and FOS overexpression attenuated the inhibitory effect of TrkB on CCL2 (Figure 5F–H). These findings suggest that c‐Fos may act as a critical intermediary in the TrkB‐mediated suppression of CCL2 expression and secretion.

To further delineate the signalling pathways through which TrkB regulates c‐Fos expression, we analysed the transcriptional profiles of TrkB‐overexpressing and control hepatocytes. KEGG pathway enrichment analysis identified potential regulatory pathways, including TNF, FoxO, and TGF‐β signalling pathway (Figure S7A). Among these, the TGFβ signalling pathway garnered particular attention due to the synergistic role of its downstream effectors, Smads, and c‐Fos in transcriptional regulation [20, 21]. This finding aligns with our previous studies in HSCs, where TrkB was shown to inhibit the TGFβ/SMADs signalling pathway. Additionally, through predictions using the JASPAR database, we found that SMAD2/3 has specific binding sites in the promoter region of the FOS gene. Specifically, SMAD2 exhibits a high‐affinity binding site at nucleotides 1041–1050 (Relative score = 0.88), while SMAD3 is predicted to have a binding site at nucleotides 1351–1360 (Relative score = 0.77) (Figure S7B). Based on these observations, we hypothesized that TrkB overexpression in hepatocytes may regulate c‐Fos through the TGFβ/SMADs signalling pathway. Further investigation into the relationship between TrkB and TGFβ/SMADs signalling revealed that PA treatment elevated TGFβ and SMAD2/3 levels, leading to increased phosphorylation of p‐SMAD2/3 and enhanced nuclear translocation (Figure S7C,D). Conversely, TrkB overexpression reduced both SMAD3 expression and phosphorylation. However, TrkB did not exert a similar effect on SMAD2 expression or phosphorylation (Figure S7E). Additionally, TrkB overexpression mimicked the effects of the p‐SMAD3 inhibitor SIS3 by decreasing inflammatory cytokine secretion in PA‐treated hepatocytes (Figure S7F) and further inhibiting HSC activation (Figure S7G). These results underscore the importance of the TGF‐β/SMAD3 pathway in TrkB‐mediated regulation, supporting its role in the suppression of liver fibrosis.

To determine whether FOS mediates TrkB regulation of the TGFβ/SMAD3/CCL2 signalling axis, we performed ChIP‐qPCR analyses. Our results showed that in PA‐treated normal hepatocytes, p‐SMAD3 binds specifically to the FOS promoter, but not to the CCL2 promoter (Figure 5I). Interestingly, TrkB overexpression significantly reduced the binding of p‐SMAD3 to the FOS promoter, but not the CCL2 promoter (Figure 5J,K). To further investigate the underlying regulatory mechanisms, we assessed the binding of H3K27ac, a marker of active transcription, to the FOS promoter and observed a significant reduction following TrkB overexpression (Figure 5L). These results suggest that TrkB overexpression disrupts the interaction between p‐SMAD3 and the FOS promoter, thereby inhibiting FOS transcriptional activation.

Research has shown that the CCL2 promoter contains binding sites for AP‐1, with FOS functioning as a subunit of AP‐1 that may directly regulate CCL2 transcription. Using ChIP‐qPCR and luciferase reporter assays, we confirmed that FOS binds to the CCL2 promoter region, and that TrkB overexpression reduces CCL2 transcriptional activity in hepatocytes (Figure 5M,N). Collectively, these findings reveal that TrkB overexpression inhibits the nuclear translocation of p‐SMAD3, thereby reducing its binding to the FOS promoter and suppressing FOS transcription. This, in turn, decreases CCL2 transcription and secretion in hepatocytes, highlighting the critical role of the TGFβ/SMAD3/FOS/CCL2 signaling axis in mediating the hepatocyte‐specific therapeutic effects of TrkB on fibrosis progression.

## 3 Discussion

Liver fibrosis is a critical precursor to the progression of chronic liver disease, potentially advancing to end‐stage conditions such as cirrhosis and liver cancer [22]. The absence of effective pharmacological treatments to cure or reverse liver fibrosis underscores the urgent need for novel anti‐fibrotic therapies. Building on our previous research, which demonstrated that TrkB inhibits HSCs' activation and proliferation by disrupting SMAD2/3 phosphorylation and nuclear translocation, we further investigated TrkB expression in hepatocytes, the primary liver cells and the initial site of fibrosis development. Our findings reveal that TrkB overexpression in hepatocytes significantly reduces the secretion of CCL2 and other pro‐fibrotic factors by inhibiting the TGFβ/SMAD3 pathway and FOS activity. This suppression not only ameliorates the inflammatory and fibrogenic microenvironment but also reduces MoMFs' recruitment, underscoring the pivotal role of hepatocyte‐specific TrkB in modulating MASH‐related fibrosis. These findings revealed the cell‐type‐specific regulatory function of TrkB in liver fibrosis, suggesting that interventions targeting hepatocyte TrkB signalling may offer a promising avenue to mitigate MASH‐related fibrosis.

To validate our findings, we adopted a multi‐faceted methodological strategy that combined both in vivo and in vitro models, including 2D and 3D systems. Our in vivo models offered a broad perspective on macrophage recruitment and fibrotic changes in a pathological context. Concurrently, the in vitro models allowed for in‐depth examination of cell‐specific TrkB overexpression and the interactions among different cell populations. This integrated approach not only strengthened the relevance of our results but also provided a clearer understanding of the role of TrkB in macrophage recruitment and fibrosis, facilitating a comprehensive analysis of these mechanisms.

We identified a novel pathway through which hepatocyte TrkB modulates liver fibrosis. TrkB notably reduces CCL2 expression and secretion, which in turn decreases the recruitment of MoMFs, acting via the TGFβ/SMAD3/FOS/CCL2 signalling axis. FOS, an intracellular transcription factor, plays a crucial role in this mechanism by forming a heterodimer with Jun family proteins to form AP‐1 [23]. Previous studies have established that TGFβ regulates RANKL‐induced osteoclastogenesis by interacting with SMAD2/3 and c‐Fos, while also modulating cytokine‐induced AP‐1 DNA binding in a SMAD3‐dependent fashion to suppress CCL2 expression [24, 25]. Our research builds on these findings, showing that phosphorylated SMAD3 directly binds to the FOS promoter, regulating its transcription—a process effectively blocked by TrkB. These insights provide a deeper understanding of the role of TrkB in fibrosis and highlight the centrality of the TGFβ/SMAD3/FOS/CCL2 pathway in disease progression.

The pathogenesis of hepatic fibrosis, as classically understood, involves a cascade of events initiated by hepatocyte damage and inflammatory signalling. This cascade converges on the activation of HSCs, which are widely regarded as the master regulator and primary effector cell in the fibrotic process [2]. Our findings provide compelling evidence that TrkB overexpression in hepatocytes attenuates fibrosis by quenching the TGFβ/SMAD3/FOS/CCL2‐dependent chemokine signal at its source, thereby alleviating the inflammatory milieu and paracrine activation of HSCs. In contrast, TrkB overexpression in HSCs directly counteracts TGFβ‐mediated pro‐fibrogenic signalling, blunting their activation and ECM production. This reveals that a dual‐targeting strategy aimed at both hepatocytes and HSCs could yield potent synergistic anti‐fibrotic effects. Activated HSCs exacerbate liver fibrosis by secreting additional TGF‐β and pro‐inflammatory factors, which in turn amplify hepatocyte injury and inflammatory signalling, thereby establishing a self‐perpetuating cycle. Simultaneous enhancement of TrkB signalling in both hepatocytes and HSCs disrupts this vicious cycle at multiple critical nodes. This combined strategy inhibits the upstream ‘driver’ signal in hepatocytes while desensitizing the downstream ‘effector’ HSCs, producing a synergistic anti‐fibrotic effect superior to targeting either cell type alone, offering a compelling novel therapeutic paradigm.

Furthermore, our findings also highlight the crucial role of macrophage infiltration in the therapeutic efficacy of TrkB for liver fibrosis. Since macrophage polarization is pivotal to fibrosis progression, investigating how TrkB regulates this process could unlock new insights into its therapeutic potential. TrkB, when activated by its ligand BDNF (brain‐derived neurotrophic factor), engages multiple intracellular signalling pathways. Studies have demonstrated that BDNF binding to TrkB activates the ERK1/2 and Akt pathways, which support the survival of specific macrophage subpopulations and aid in myocardial infarction repair [26]. Additionally, increased BDNF levels lead to greater TrkB phosphorylation, activating the CREB/BDNF/TrkB signalling pathway and promoting M2 macrophage polarization with anti‐inflammatory effects, particularly in diabetic atherosclerosis [27]. Future research should investigate whether TrkB overexpression can effectively modulate macrophage polarization to enhance therapeutic approaches for liver fibrosis, thereby providing a deeper understanding of its role in fibrosis management.

Looking ahead, the BDNF/TrkB signalling pathway shows considerable promise as a therapeutic target for addressing fibrosis across multiple organs. Its key role in regulating fibroblast activation and managing inflammatory responses makes it central to fibrosis development in the liver, lungs, heart, and kidneys [28, 29, 30]. For instance, in pulmonary fibrosis, the BDNF/TrkB pathway worsens the disease by promoting epithelial‐to‐mesenchymal transition (EMT) [31]. Our findings, which demonstrate the anti‐fibrotic effects of TrkB overexpression in hepatocytes via the TGFβ/SMAD3/FOS/CCL2 axis, suggest that similar mechanisms may be at play in other organs as well. This insight expands the potential applications of targeting TrkB for the treatment of various fibrotic conditions, opening new opportunities for therapeutic intervention and further research.

In summary, our research elucidates the functional role of hepatocyte TrkB in liver fibrosis through regulation of the TGFβ/SMAD3/FOS/CCL2 signalling axis. This regulatory mechanism contributes to the modulation of both fibrotic progression and immune microenvironment remodelling. The findings reveal complex cellular crosstalk between hepatocytes, HSCs, and MoMFs coordinated by TrkB signalling, providing new insights into the molecular pathways underlying fibrotic liver disease.

## 4 Materials and Methods

### 4.1 Animals and Treatment

All animal experiments adhered to protocols approved by the Institutional Animal Care and Use Committee (IACUC) of Zhongshan Hospital, Fudan University. Male ob/ob mice (6–8 weeks old) were purchased from JSJ Company (Shanghai, China). At 8 weeks of age, the mice were placed on a Gubra‐Amylin MASH (GAN) diet (D09100310, Research Diets, NJ, USA) for 16 weeks. AAV8 vectors were administered via tail vein injection at weeks 8 and 12, with a dose of 1 × 10^11^ vector genomes per gram (VG/g). Liver samples were collected at the conclusion of each experiment.

### 4.2 Patients and Sample Collection

Eighteen human liver fibrosis specimens were collected at Zhongshan Hospital, Fudan University. Informed consent was obtained from all participants prior to enrollment. The study was approved by the Institutional Ethics Committee of Zhongshan Hospital, Fudan University (approval no. B2023‐270) and conducted in accordance with the Declaration of Helsinki. All clinical specimens were obtained from enrolled patients.

### 4.3 Histological Staining

Liver samples were fixed in 4% paraformaldehyde (Sigma‐Aldrich) for 20 min and processed using standard staining protocols. Haematoxylin and eosin (H&E) staining and Sirius Red staining were performed on paraffin‐embedded liver sections to examine liver morphology and evaluate fibrosis stages. Hepatic fibrosis was quantitatively analysed using ImageJ software in a blinded manner based on immunohistochemical visualization. The antibodies used for immunohistochemical staining are detailed in Table S1.

### 4.4 Cell Culture and Treatment

Hepatoma HepG2 cells (serial no. SCSP‐510) were obtained from the National Collection of Authenticated Cell Cultures, and human hepatic stellate LX2 cells (serial no. SCC064) were purchased from Millipore. Both HepG2 and LX2 cells were cultured in DMEM supplemented with 10% fetal bovine serum (FBS) under a 5% CO_2_ atmosphere at 37°C. For palmitic acid (PA) treatment, cells were exposed to 0.5 mM PA (Sigma, P0500) for 48 h.

### 4.5 Stable Cell Line Construction

To generate a stable *TrkB*‐overexpressing cell line, *TrkB* cDNA was inserted into the *EcoRI* and *BamHI* sites of a pHBLV vector. For *TrkB* knockdown, a *shTrkB* sequence targeting the *TrkB* coding sequence (CDS) was designed and cloned into the *EcoRI* and *BamHI* sites of an LV3 vector. The constructs were co‐transfected with *pMD2G* and *psPAX2* into HEK293T cells, and lentivirus was harvested after 48–72 h. The lentivirus was used to infect HepG2 cells in the presence of polybrene (final concentration: 8 μg/mL). Stable overexpression or knockdown cells were selected using 2 μg/mL puromycin. Primer and shRNA sequences are detailed in Table S2.

### 4.6 Plasmid Construction

The full‐length cDNA of human TrkB was amplified by PCR and cloned into the pLVX‐GFP vector, while human FOS and MYC cDNAs were cloned into the pcDNA3.1 vector (Life Technologies). shRNAs targeting FOS and MYC (two oligos per gene) were designed using the Sigma website (https://www.sigmaaldrich.cn/CN/zh) and inserted into the pLKO.1‐BFP vector. Primer sequences for plasmid construction are provided in Table S1. Cell transfection was conducted using Lipofectamine 3000 (Invitrogen) following the manufacturer's protocol. For lentivirus production, the above lentiviral plasmids were co‐transfected with packaging plasmids (pMDLg/pRRE, pRSV‐Rev, and pCMV‐VSV‐G) into HEK293T cells. After 48 h, the supernatants were filtered using a 0.45 μm filter and collected to infect HepG2 cells for 48 h. Stable GFP‐ or BFP‐expressing HepG2 cells were subsequently sorted by flow cytometry.

### 4.7 Quantitative Reverse Transcriptase Polymerase Chain Reaction (qRT‐PCR)

Total RNA was extracted by TRIZOL reagent. Hifair II 1st Strand cDNA Synthesis SuperMix (11123ES60, Yeasen, Shanghai, China) was applied to synthesize complementary DNA (cDNA). mRNA levels were normalized to GAPDH. Hieff qPCR SYBR Green Master Mix (11202ES03, Yeasen, Shanghai, China) was used for PCR amplification, and relative gene expression level was calculated using a 2^−ΔΔ*CT*^ method. All the primers used in this study were listed in Table S2.

### 4.8 Western Blot Assay

Total protein was extracted from cultured cells, and mouse liver samples using RIPA lysis buffer added with protease inhibitor cocktail tablets. Stock solution (25×) of protease inhibitor cocktail tablets were prepared from Roche (cat# 04693132001). Then, BCA protein quantification kit (20201ES76, Yeasen, Shanghai, China) was used to measure protein concentration. Next, 10% polyacrylamide gel was produced to separate each protein, and separated proteins were transferred into PVDF membranes (Millipore, IPVH00010). After blocking with protein‐free rapid blocking buffer (PS108, Yamei, Shanghai, China) for 15 min at room temperature, the membranes were incubated with a primary antibody overnight at 4°C, and then a corresponding secondary antibody for 60 min at room temperature. The ChemiDoc MP Imaging System (Bio‐Rad) was used for imaging of blots. All results were performed in three independent experiments. Primary and secondary antibodies were listed in Table S1.

### 4.9 Enzyme‐Linked Immunosorbent Assay (ELISA)

ELISA was performed to quantify the concentration of IL‐6, TNFα, CCL2, and TGFβ in the cell culture supernatant according to the manufacturer's protocol for the ELISA kit (Shanghai Hengyuan biological co., HS1057‐Mu, HS051‐Mu, HS459‐Mu, HS1349‐Mu, HB1946‐Hu, HB090‐Hu, HB2328‐Hu, HB2446‐Hu).

### 4.10 CHIP‐qPCR

Crosslink the HepG2‐GFP/TrkB cells on ice with 1% formaldehyde, terminated with 0.125 M glycine. After washing, use DNA ultrasonic disruptor (Covaris E220) to break the chromatin DNA, producing DNA fragments of 200–500 bp. Immunoprecipitation was performed using equal amounts of cut DNA with Rabbit c‐FOS antibody (CST, #31254) or Rabbit Phospho‐SMAD3 (Ser423/425) antibody (CST, #9520), and Rabbit IgG (CST, #3900) for 6 h. Then incubate the immunoprecipitate with protein G magnetic beads overnight, wash and collect the DNA‐antibody‐magnetic beads complex for de crosslinking. Cut DNA without immunoprecipitation was used as input control. The recovered DNA was subjected to 40 cycles qPCR using the designed ChIP‐qPCR primers. The ChIP–qPCR primers used are shown in Table S1.

### 4.11 Dual‐Luciferase Reporter Assay

Insert the human FOS promoter (chr14:75278368–75278871) or CCL2 promoter region (chr17:34254912–34255345) into the pGL4.11 firefly luciferase plasmid vector. Primers for pGL4.11 plasmids construction were listed in Table S1. HepG2‐GFP/TrkB cell lines were seeded in 96‐well black plates, then co‐transfected with 0.1 μg pGL4.11 FOSpro/CCL2pro plasmid and 0.001 μg pGL4.75 renilla luciferase plasmid. After 48 h of incubation, the cell lysate supernatant was harvested and luciferase activity was measured using the Dual‐Luciferase Kit (Promega, Madison, MI). Firefly luciferase values are standardized to renilla luciferase values.

### 4.12 Isolation of Primary Mouse Hepatocyte

At 16th week of GAN feeding, primary mouse cells were isolated using the collagenase perfusion method. After perfusion, the liver was removed and cells were isolated in a 70 μm filter membrane with 1640 medium and filtered. After centrifuging at 50 g for 2 min at 4°C, the pellet contained hepatocytes and the supernatant containing non‐parenchymal cells were collected separately. Cells were stained with Trypan Blue and counted by blood counting chamber.

### 4.13 Transcriptome Sequencing and Analysis

Total RNA was extracted and purified using RNAiso Plus (TaKaRa, Code No.: 9109, Japan), and then RNA amount was measured by NanoDrop 2000 (Thermo Fisher Scientific, US). Following mRNA enrichment, SuperScript II reverse transcriptase (Invitrogen, cat. 1896649, USA) was applied to create cDNA. After adding ‘A tailing’, purified cDNA products were amplified by the following PCR program: 3 min at 95°C, 8 cycles of 98°C for 15 s, 60°C for 15 s, 72°C for 30 s and final extension at 72°C for 5 min. Then the paired‐end sequencing was performed in an illumina Novaseq 6000 (LC‐Bio Technology CO. Ltd., Hangzhou, China). HISAT2 was used to map reads against the reference genome. StringTie was used to value expression levels of all mRNAs by calculating the fragments per kilobase per millon (FPKM). The R package edgeR was performed to select the differentially expressed genes (https://bioconductor.org/packages/release/bioc/html/edgeR.html). The sequencing datasets were submitted to the Sequence Read Archive (SRA) data set with registration number PRJNA.

### 4.14 Single‐Cell RNA Sequencing

The scRNA‐seq was performed on FACS‐enriched live CD45+ cells from the livers of GAN‐fed ob/ob mice. The scRNAseq data were preprocessed using Cell Ranger Suite (v3.1.0, https://support.10xgenomics.com) with refdata‐cellranger‐mm10–3.0.0 transcriptome as a reference to map reads on mouse genome (mm10) using STAR. Low‐quality cells with fewer than 200 genes with reads and cells with more than 10% mitochondrial content were filtered out. Cell clustering, marker identification, and visualization were performed using Seurat v4. The R package SingleR was used to determine cell types of the clusters using the ImmGen dataset as a reference for cell‐specific gene signatures. Further resolution of cell types was carried out using known gene markers associated with different immune cell subtypes. Genes differentially expressed between AAV8‐GFP and AAV8‐TrkB samples within clusters of interest were identified using a nonparametric Wilcoxon rank sum test, and statistically significant genes (FDR < 0.05 and fold cutoffs when specified) were subjected to enrichment analysis using QIAGEN's IPA software (QIAGEN, Redwood City; www.qiagen.com/ingenuity). The sequencing datasets were submitted to the Sequence Read Archive (SRA) data set with registration number PRJNA.

### 4.15 3D Liver NACs Model Construction

In this study, we employed a 3D liver NACs model construction strategy utilizing nucleic acid nanomaterials (NAC‐Linkers) [26], comprising three distinct liver models. The 3D steatosis model was generated using primary hepatocytes as the sole cell type, while the 3D MASH model incorporated both primary hepatocytes and hepatic stellate cells. Additionally, the PBMC immune infiltration model was established by introducing live‐cell fluorescently labelled PBMCs into the culture supernatant of the 3D MASH model. All 3D liver NACs models contained a total of 3000 cells per NACs model, with a hepatocyte‐to‐stellate cell ratio of 10:1 to mimic the physiological cell composition of the liver.

### 4.16 Statistical Analysis

All data were expressed as mean ± standard error of mean (SEM). Statistical analysis was performed with SPSS 20.0 software. When two groups were compared, the student *t*‐test was used after normal distribution test. If normality or homogeneity of variance is not met, Mann–Whitney *U* test is used to compare between the two groups. When the experimental design involves more than 2 groups, the analysis of variance (ANOVA) was used to compare between groups after normal distribution test, and multiple comparisons between two given groups were completed by the least significant difference (LSD) test. If normality or homogeneity of variance is not met, Kruskal‐Walli's test is used to compare between groups, and multiple comparisons between two given groups were completed by Hodges‐Lehmann test. A value of *p* < 0.05 was considered to be statistically significant. *p* values are shown as *p < 0.05, **p < 0.01, ***p < 0.001 and ****p < 0.0001.

## Author Contributions

L.D., Q.Y., C.Z., G.S. and X.S. provided the concept, designed the study and provided the conceptual framework for the study; L.D., Q.Y. and C.Z. provided the concept and the financial support; Y.C., J.W. and S.L. performed all experiments and analysed the data; Y.C. and J.W. wrote the manuscript with the help of K.Y. and S.L.; Y.C., J.W., K.Y. and S.L. contributed with manuscript preparation and finalization. H.W. provided DNA Origami for 3D liver spheroids. Y.Z., S.W., L.D., Q.Y., G.S., C.Z. and X.S. provided conceptual evaluation of the project. All authors commented on and approved the manuscript. Authors thank Yu Song for providing lentivirus and transfection reagents, and helpful comments on this manuscript.

## Funding

This work was supported by National Science Foundation of China, 82570725, 81700550; Shanghai Science and Technology Committee, 20ZR1411200, 22140900700, 23ZR1411300, 24YF2704400.

## Conflicts of Interest

G.S. is an employee of Puheng Technology Co. Ltd. The other authors declare no conflicts of interest.

## Supporting information


**Figure S1:** Spearman correlation analysis of Sirius Red staining and TrkB H‐scores in MASH patients.
**Figure S2:** Schematic overview of the supernatant transfer experiments and its effects on LX2 cell activation and proliferation.
**Figure S3:** qRT‐PCR analysis of inflammatory and fibrotic gene expression in hepatocytes and LX2 cells.
**Figure S4:** Co‐immunofluorescence staining of CCR2 and CD68 in liver samples from MASH patients with mild and severe fibrosis.
**Figure S5:** Validation of CCL2 expression in primary hepatocytes and 3D liver culture supernatants.
**Figure S6:** qRT‐PCR analysis of *CCL2* in FOS and MYC overexpressing HepG2 cells.
**Figure S7:** KEGG pathway enrichment analysis and validation of the TGFβ/SMAD signalling pathway regulating FOS expression.
**Table S1:** List of antibodies used for Western blot and immunofluorescence.
**Table S2:** Sequences of primers used for qRT‐PCR, molecular cloning, ChIP‐qPCR, and Luciferase Reporter Assay.
**Table S3:** Sequences of shRNAs used for gene knockdown.

## Data Availability

All data are available in the main text or the Supporting Information.
