# Supplementary material for: Hepatocyte TrkB Acts as a Gatekeeper Against MASH‐Related Liver Fibrosis by Suppressing the TGFβ/CCL2 Axis and Macrophage Infiltration
Source: Cell Prolif. 2026 Apr 3;59(8):e70202. doi: 10.1111/cpr.70202 (PMC13325918; doi:10.1111/cpr.70202)
Supplement: Supplementary file 1 [file CPR-59-e70202-s001.docx]

Supplementary Materials for

**Hepatocyte TrkB acts as a gatekeeper against MASH-related liver fibrosis by suppressing the TGFβ/CCL2 axis and macrophage infiltration**

Yueying Chen *et al.*

Corresponding author: Guangqi Song, Qunyan Yao and Ling Dong, dong.ling@zs-hospital.sh.cn; [yao.qunyan@zs-hospital.sh.cn](mailto:yao.qunyan@zs-hospital.sh.cn); zhuchangfeng@fudan.edu.cn

**This PDF file includes:**

Figs. S1 to S7

Tables S1 to S3

**Figures and Tables**

**Figure S1**

(A) Spearman correlation analysis of the positive area of Sirius Red staining and H-score values of TrkB in MASH patients.

**Figure S2**

(A) Schematic overview of the supernatant transfer experiments. HepG2 and HepG2-TrkB were treated with PA (0.5 mM) for 48 h followed by incubation with fresh DMEM for additional 24 h. Then the conditional medium was collected and transferred to incubate LX2. **(B-C)** Protein levels of COL1A1 and αSMA in LX2 cells as determined by Western blot analysis. Protein levels of COL1A1 and αSMA in LX2 cells as determined by Western blot analysis. **(D)** Cell proliferation evaluation of LX2 cells incubated with the conditional medium from control and PA-treated HepG2 and HepG2-TrkB cells using the CCK8 assay. n = 5 per group. Data were mean ± SEM (*p <0.05, **p <0.01, ***p <0.001; One way ANOVA). Schematic illustration (A) was created by biorender.

**Figure S3**

**(A)** qRT-PCR analysis of *IL6, TNFA, CCL2* and *TGFB* in control and PA-treated HepG2 and HepG2-TrkB (n = 4 per group). **(B)** qRT-PCR analysis of *COL1A1, ACTA1, TGFB1, TIMP1* and *PDGFRB* in LX2 cells (n = 5 per group). Data were mean ± SEM (*p <0.05, **p <0.01, ***p <0.001; One way ANOVA).

**Figure S4**

**(A)** CCR2 and CD68 co-immunofluorescence in liver samples from mild (S0-1) and severe fibrosis (≥S2) MASH patients. Scale bars, 20 µm. **(B)** Quantification of CD68 and CCR2 positive area in (A). Data were mean ± SEM (*p <0.05, **p <0.01, ***p <0.001; Student’s *t* test).

**Figure S5**

(A) qRT-PCR analysis of *Ccl2* in primary hepatocytes from GAN-fed ob/ob of AAV8-control and AAV8-TrkB mice (n = 5 per group). **(B)** ELISA analysis of CCL2 concentration in the supernatant of 3D livers (n = 3 per group). Data were mean ± SEM (*p <0.05, **p <0.01, ***p <0.001; Student’s *t* test in A; One way ANOVA inB).

**Figure S6**

**(A)** qRT-PCR analysis of *CCL2* in *FOS* and *MYC* overexpressed-HepG2 cells (n = 3 per group). Data were mean ± SEM (*p <0.05, **p <0.01, ***p <0.001; Student’s *t* test).

**Figure S7**

**(A)** KEGG pathway enrichment analysis of DEGs between control and TrkB-overexpressing hepatocytes (n=5). **(B)** Prediction map of SMAD2 (upper panel) and SMAD3 (lower panel) binding sites on the FOS promoter region. **(C)** Western blot analysis of SMAD2/3, pSMAD2/3 and TGFβ in HepG2 cells treated with or without PA (0.5 mM) for 48 h. **(D)** Western blot analysis of SMAD2/3 and pSMAD2/3 in the cytoplasm and nucleus of HepG2 cells treated with or without PA (0.5 mM) for 48 h. **(E)** Representative images of Western blot analysis of SMAD2/3 and Psmad2/3 in HepG2 and HepG2-TrkB cells treated with or without PA (0.5 mM) for 48 h. **(f)** ELISA analysis of IL-6, TNFα, CCL2 and TGFβ concentrations in HepG2 and HepG2-TrkB cells treated with or without SIS3 (10 μM) for 6 h. **(g)** Western blot analysis of COL1A1 and α-SMA in LX2 cells incubated with the conditional medium from HepG2 and HepG2-TrkB cells treated with or without SIS3 (10 μM) for 6 h. Data were mean ± SEM (*p <0.05, **p <0.01, ***p <0.001; One way ANOVA).

**Supplementary Table 1. Antibodies**

| **Name** | **Species reativity** | **Application and dilution ratio** | **Supplier** | **Cat No.** |
| --- | --- | --- | --- | --- |
| Anti-TrkB | Human, Mouse | WB (1:1000) | abcam | ab18987 |
| Anti-TrkB | Human, Mouse | IF (1:50); IHC (1:50) | Santa Cruz | sc-377218 |
| Anti-Phospho-Smad3 | Human, Mouse | WB (1:1000); CHIP (1:100) | CST | 9520 |
| Anti-Smad3 | Human, Mouse | WB (1:1000) | CST | 9523 |
| Anti-COL1A1 | Human, Mouse | WB (1:1000) | ABclonal | A16891 |
| Anti-α-SMA | Human, Mouse | WB (1:1000) | ABclonal | A17910 |
| Anti-α-SMA | Human, Mouse | IF (1:50); IHC (1:500) | abcam | ab179467 |
| Anti-TGFβ | Human, Mouse | WB (1:1000) | Proteintech | 21898-1-AP |
| Anti-FOS | Human, Mouse | WB (1:1000); CHIP (1:50) | CST | 2250 |
| Anti-MCP1 | Human, Mouse | WB (1:1000) | Proteintech | 26161-1-AP |
| Anti-Lamin | Human, Mouse | WB (1:5000) | Proteintech | 12987-1-AP |
| Anti-Tubulin | Human, Mouse | WB (1:5000) | Proteintech | 11224-1-AP |
| Anti-GAPDH | Human, mouse | WB (1:100000) | Thermo Fisher | 14-9523-82 |
| Anti-F4/80 | mouse | IF (1:150) | CST | 70076 |
| Anti-CD11B | mouse | IF (1:100) | abcam | ab133357 |
| Anti-CCR2 | Human, mouse | IF (1:100) | abcam | Ab273050 |
| Anti-CD68 | Human | IF (1:100) | abcam | ab955 |

**Supplementary Table 2. Primers**

1. Primers for qRT-PCR

| **Name** | **Sequence** | **Supplier** |
| --- | --- | --- |
| mTrkB-F | CCCAGGGAAGAGTCCTTCAG | TSINGKE |
| mTrkB-R | GATGTTCTTCCGGGTGTGTG | TSINGKE |
| mCol1a1-F | TGACTGGAAGAGCGGAGAGT | TSINGKE |
| mCol1a1-R | GTTCGGGCTGATGTACCAGT | TSINGKE |
| mCol3a1-F | CTGTAACATGGAAACTGGGGAAA | TSINGKE |
| mCol3a1-R | CCATAGCTGAACTGAAAACCACC | TSINGKE |
| mTGFβ1-F | CCACCTGCAAGACCATCGAC | TSINGKE |
| mTGFβ1-R | CTGGCGAGCCTTAGTTTGGAC | TSINGKE |
| mCTGF-F | GGCCTCTTCTGCGATTTCG | TSINGKE |
| mCTGF-R | GCAGCTTGACCCTTCTCGG | TSINGKE |
| mACTA2-F | CCCAGACATCAGGGAGTAATGG | TSINGKE |
| mACTA2-R | TCTATCGGATACTTCAGCGTCA | TSINGKE |
| mIL1b-R | GCAACTGTTCCTGAACTCAACT | TSINGKE |
| mIL1b-F | ATCTTTTGGGGTCCGTCAACT | TSINGKE |
| mIL6-F | TAGTCCTTCCTACCCCAATTTCC | TSINGKE |
| mIL6-R | TTGGTCCTTAGCCACTCCTTC | TSINGKE |
| mTNFα-F | CCCTCACACTCAGATCATCTTCT | TSINGKE |
| mTNFα-R | GCTACGACGTGGGCTACAG | TSINGKE |
| mCCL2 -F | TTAAAAACCTGGATCGGAACCAA | TSINGKE |
| mCCL2 -R | GCATTAGCTTCAGATTTACGGGT | TSINGKE |
| mGAPDH-F | AGGTCGGTGTGAACGGATTTG | TSINGKE |
| mGAPDH-R | TGTAGACCATGTAGTTGAGGTCA | TSINGKE |
| hTrkB-F | ACAGTCAGCTCAAGCCAGACAC | TSINGKE |
| hTrkB-R | GTCCTGCTCAGGACAGAGGTTA | TSINGKE |
| hGAPDH-F | AACAGCCTCAAGATCATCAG | TSINGKE |
| hGAPDH-R | AGTCCTTCCACGATACCAA | TSINGKE |
| hα-SMA-F | TGCCTTGGTGTGTGACAATG | TSINGKE |
| hα-SMA-R | TTGTCCCATTCCCACCATCA | TSINGKE |
| hTGFβ-F | CAGCAGGGATAACACACTGC | TSINGKE |
| hTGFβ-R | CATGAGAAGCAGGAAAGGCC | TSINGKE |
| hCol1a1-F | TGACCTCAAGATGTGCCACT | TSINGKE |
| hCol1a1-R | ACCAGTCTCCATGTTGCAGA | TSINGKE |
| hTIMP1-F | GGAGAGTGTCTGCGGATACTTC | TSINGKE |
| hTIMP1-R | GCAGGTAGTGATGTGCAAGAGTC | TSINGKE |
| hPDGFβ-F | CATGGGGGTATGGTTTTGTC | TSINGKE |
| hPDGFβ-R | GTAAGGTGCCAACCTGCAAT | TSINGKE |
| hIL6-F | GAGTAGTGAGGAACAAGCCAGA | TSINGKE |
| hIL6-R | AAGCTGCGCAGAATGAGATGA | TSINGKE |
| hTNFα-F | TGGCGTGGAGCTGAGAGATA | TSINGKE |
| hTNFα-R | TGATGGCAGAGAGGAGGTTG | TSINGKE |
| hFOS-F | GCCTCTCTTACTACCACTCACC | TSINGKE |
| hFOS-R | AGATGGCAGTGACCGTGGGAAT | TSINGKE |
| hMYC-F | CCTGGTGCTCCATGAGGAGAC | TSINGKE |
| hMYC-R | CAGACTCTGACCTTTTGCCAGG | TSINGKE |
| hCDKN1A-F | AGGTGGACCTGGAGACTCTCAG | TSINGKE |
| hCDKN1A-R | TCCTCTTGGAGAAGATCAGCCG | TSINGKE |
| hSTAT1-F | ATGGCAGTCTGGCGGCTGAATT | TSINGKE |
| hSTAT1-R | CCAAACCAGGCTGGCACAATTG | TSINGKE |
| hCCL2-F | AGAATCACCAGCAGCAAGTGTCC | TSINGKE |
| hCCL2-R | TCCTGAACCCACTTCTGCTTGG | TSINGKE |

2. Primers for molecular cloning

| **Name** | **Sequence** | **Supplier** |
| --- | --- | --- |
| pCDNA3.1-FOS-F | GAGACCCAAGCTTGGTACCATGATTCTCGGGCTTCAA | TSINGKE |
| pCDNA3.1-FOS-R | CCATGGTGGCGGATCCATTATCGTCGTCATCCTTGTAAT CAGGGCCAGCAGCGTG | TSINGKE |
| pCDNA3.1-MYC-F | GAGACCCAAGCTTGGTACCCTGGATTTTTTTTCGGGTAGT | TSINGKE |
| pCDNA3.1-MYC-R | GCCATGGTGGCGGATCCCTTATCGTCGTCATCCTTGTAATCCGCACAAAGAGTTCCGTAG | TSINGKE |

3. Primers for ChIP-qPCR

| **Name** | **Sequence** | **Supplier** |
| --- | --- | --- |
| ChIP-F-FOSpro | CGAAACCCCTCATCTTGGGG | TSINGKE |
| ChIP-R-FOSpro | GAGTGTAAACGTCACGGGCT | TSINGKE |
| ChIP-F-CCL2pro | AGTCTGGGCTTAATGGCACC | TSINGKE |
| ChIP-R-CCL2pro | AGTCAAGCAGGAGGAGGGAT | TSINGKE |

4. Primers for Luciferase Reporter Assay

| **Name** | **Sequence** | **Supplier** |
| --- | --- | --- |
| FOSpro-pGL4-Kpn1F | CTAACTGGCCGGTACCCCAGGTGCGAATGTTCTC | TSINGKE |
| FOSpro-pGL4-Kpn1R | TCTTGATATCCTCGAGAACGTCACGGGCTCAACC | TSINGKE |
| Ccl2pro-pGL4-Kpn1F | CTAACTGGCCGGTACCAGGTAAGCTGGCAGCGAG | TSINGKE |
| Ccl2pro-pGL4-Kpn1R | TCTTGATATCCTCGAGGCGAGAGTGCGAGCTTCA | TSINGKE |

**Supplementary Table 3. Sequences for *shRNAs***

| **Name** | **Sequences (5’ to 3’)** | **Supplier** |
| --- | --- | --- |
| scramble shRNAF | CCGGCCTAAGGTTAAGTCGCCCTCGCTCGAGCGAGGGCGACTTAACCTTAGGTTTTTG | TSINGKE |
| scramble shRNAR | AATTCAAAAACCTAAGGTTAAGTCGCCCTCGCTCGAGCGAGGGCGACTTAACCTTAGG | TSINGKE |
| shMYC #1shRNAF | CCGGCCTGAGACAGATCAGCAACAACTCGAGTTGTTGCTGATCTGTCTCAGGTTTTTG | TSINGKE |
| shMYC #1shRNAR | AATTCAAAAACCTGAGACAGATCAGCAACAACTCGAGTTGTTGCTGATCTGTCTCAGG | TSINGKE |
| shMYC #2shRNAF | CCGGCAGTTGAAACACAAACTTGAACTCGAGTTCAAGTTTGTGTTTCAACTGTTTTTG | TSINGKE |
| shMYC #2shRNAR | AATTCAAAAACAGTTGAAACACAAACTTGAACTCGAGTTCAAGTTTGTGTTTCAACTG | TSINGKE |
| shFOS #1shRNAF | CCGGGCGGAGACAGACCAACTAGAACTCGAGTTCTAGTTGGTCTGTCTCCGCTTTTTG | TSINGKE |
| shFOS #1shRNAR | AATTCAAAAAGCGGAGACAGACCAACTAGAACTCGAGTTCTAGTTGGTCTGTCTCCGC | TSINGKE |
| shFOS #2shRNAF | CCGGTCTGCTTTGCAGACCGAGATTCTCGAGAATCTCGGTCTGCAAAGCAGATTTTTG | TSINGKE |
| shFOS #2shRNAR | AATTCAAAAATCTGCTTTGCAGACCGAGATTCTCGAGAATCTCGGTCTGCAAAGCAGA | TSINGKE |
